# Differential impacts of carp and salmon pituitary extracts on induced oogenesis, egg quality, molecular ontogeny and embryonic developmental competence in European eel

**DOI:** 10.1371/journal.pone.0235617

**Published:** 2020-07-07

**Authors:** Johanna S. Kottmann, Michelle G. P. Jørgensen, Francesca Bertolini, Adrian Loh, Jonna Tomkiewicz

**Affiliations:** 1 National Institute of Aquatic Resources, Technical University of Denmark, Kgs. Lyngby, Denmark; 2 School of Science, University of Greenwich, Chatham Maritime, Kent, United Kingdom; National Cheng Kung University, TAIWAN

## Abstract

Low egg quality and embryonic survival are critical challenges in aquaculture, where assisted reproduction procedures and other factors may impact egg quality. This includes European eel (*Anguilla anguilla*), where pituitary extract from carp (CPE) or salmon (SPE) is applied to override a dopaminergic inhibition of the neuroendocrine system, preventing gonadotropin secretion and gonadal development. The present study used either CPE or SPE to induce vitellogenesis in female European eel and compared impacts on egg quality and offspring developmental competence with emphasis on the maternal-to-zygotic transition (MZT). Females treated with SPE produced significantly higher proportions of floating eggs with fewer cleavage abnormalities and higher embryonic survival. These findings related successful embryogenesis to higher abundance of mRNA transcripts of genes involved in cell adhesion, activation of MZT, and immune response (*dcbld1*, *epcam*, *oct4*, *igm*) throughout embryonic development. The abundance of mRNA transcripts of *cldnd*, *foxr1*, *cea*, *ccna1*, *ccnb1*, *ccnb2*, *zar1*, *oct4*, and *npm2* was relatively stable during the first eight hours, followed by a drop during MZT and low levels thereafter, indicating transfer and subsequent clearance of maternal mRNA. mRNA abundance of *zar1*, *epcam*, and *dicer1* was associated with cleavage abnormalities, while mRNA abundance of *zar1*, *sox2*, *foxr1*, *cldnd*, *phb2*, *neurod4*, and *neurog1* (before MZT) was associated with subsequent embryonic survival. In a second pattern, low initial mRNA abundance with an increase during MZT and higher levels persisting thereafter indicating the activation of zygotic transcription. mRNA abundance of *ccna1*, *npm2*, *oct4*, *neurod4*, and *neurog1* during later embryonic development was associated with hatch success. A deviating pattern was observed for *dcbld1*, which mRNA levels followed the maternal-effect gene pattern but only for embryos from SPE treated females. Together, the differences in offspring production and performance reported in this study show that PE composition impacts egg quality and embryogenesis and in particular, the transition from initial maternal transcripts to zygotic transcription.

## Introduction

The aquaculture sector has expanded rapidly and further development relies on diversification and on closing the life cycle for aquatic species in captivity [1,2]. However, high variability in egg quality and low survival during embryonic development pose a challenge to captive offspring production in fish aquaculture [3–6].

In oviparous teleosts, embryonic and early larval development is influenced by intrinsic properties of the gamete, as well as extrinsic factors, e.g. ambient conditions during egg incubation and larval culture [4,7,8]. While the influence of maternal nutrition and egg nutrient composition on offspring quality is well established, studies of embryogenesis continue to uncover vital functions of different cytoplasmic factors such as messenger RNAs (mRNAs) that also are incorporated into the developing oocyte [5,9]. Thus, these maternal mRNA transcripts deposited into the egg during oocyte development have proven to be essential drivers of zygotic and early embryonic development until the mid-blastula transition [7,9,10]. At this stage, developmental control is taken over by the embryo through transcriptional activation of the zygotic genome [11,12]. In this context, the maternal mRNA abundance and activation of the maternal-to-zygotic transition (MZT) are vital for the molecular ontogeny and development of the embryo, including the activation of zygotic transcription and the clearance of maternal mRNA [13–17].

Studies of molecular ontogeny during early embryogenesis have documented a tight relationship between the abundance of specific maternal mRNA transcripts, egg quality, and embryonic developmental competence [18–23]. Embryogenesis is mainly governed by maternal transcripts until MZT, when embryonic mRNA transcripts take over accompanied by clearance of maternal RNA. Examples of maternal transcripts include genes involved in cell cycle progression (*ccna1*, *ccnb1*, *ccnb2*, *npm2*), cell division (*foxr1*, *cea*), cell adhesion (*cdhr2*, *cldnd*, *dcbld1*, *epcam*), microRNA regulation (*dicer1*), pluripotency regulation, cell signaling, and activation of MZT (*zar1*, *oct4*, *sox2*, *phb2*) [9,10,17]. These so called maternal-effect genes often exhibit early transcript accumulation with subsequent decrease, once the related early developmental processes are completed [24–27]. Also, maternally-derived immune factors (e.g. *c3*, *igm*, *il1β*) appear to play a role in early protection and organogenesis of the embryo and larva until the adaptive immune system is fully developed [28–31]. On the other hand, genes related to processes such as organogenesis often begin transcription during later stages of the embryonic development [26,32]. This may include molecular mechanisms that regulate processes of neurogenesis (e.g. *neurod4*, *neurog1*) to establish the nervous system during embryonic stages, which to date have been mainly studied in the model species zebrafish (*Danio rerio*) [33–35].

The incorporation of these maternal transcripts into the cytoplasm occurs during oocyte development. In oviparous female teleosts, the reproductive developmental processes are regulated by gonadotropins (GTH), i.e. follicle stimulating hormone (FSH) and luteinizing hormone (LH), in combination with maturation-inducing steroid (MIS), and maturation-promoting factor (MPF) [36]. Here, the production of FSH and LH by the pituitary is regulated by the brain that also stimulates their release through the action of gonadotropin-releasing hormone (GnRH). After being released into the bloodstream, LH and FSH target the ovaries, regulating the production of sex steroids and vitellogenesis. However, these endocrine processes, which need to be triggered and sustained for successful oocyte production, may be impeded. Thus, while some fish species reproduce naturally in captivity under suitable culture conditions, the breeding capability of other is inhibited. Manipulation of extrinsic factors, such as photoperiod and temperature, is sufficient to initiate successful gametogenesis and reproduction in some species, whereas in others hormonal therapy is required. Such assisted reproduction treatments may target different regulatory mechanisms along the brain-pituitary-gonad pathway, depending on the impeded state of the reproductive cycle [6]. This hormonal manipulation of reproductive functions may impact gamete quality, thereby affecting fertilization success, molecular ontogeny, and developmental competence of the embryo.

Anguillid eels belong to the group of aquaculture species, which do not reproduce in captivity. In particular, the European eel (*Anguilla anguilla*) and the Japanese eel (*A*. *japonica*) are high-value species in aquaculture and the development of hatchery technology for closed-cycle production would be of great value. Their complex diadromous life cycle, including long migrations to their spawning area, makes this difficult. During the silvering process that marks the onset of the spawning migration, intrinsic inhibitory mechanisms at the brain-pituitary level arrest puberty at an early stage [37], with this inhibition being released when approaching the spawning area. As yet, the endocrine mechanisms remain unresolved and in captivity this pre-pubertal blockade prevents natural reproductive development and spawning. Captive offspring production requires assisted reproduction protocols, with administration of exogenous gonadotropins to induce sexual maturation and sustain gamete development [6,38]. While such assisted reproduction protocols have led to stable production of viable eggs and larvae reaching the first-feeding stage for European eel [39–41], and production of glass eels for the Japanese eel [42,43], shortcomings in egg quality and offspring survival persist with a major bottleneck during the embryonic development [44].

For female eels, assisted reproduction protocols commonly use pituitary extracts (PE) from carp (CPE) or salmon (SPE) as source of FSH and LH to induce vitellogenesis, while follicular maturation is completed by administration of a MIS, e.g. 17α,20ß-dihydroxy-4-pregnen-3-one (DHP) [38,45]. The protocols using PE to induce vitellogenesis have varied greatly over time, both regarding product and dose. The first successful treatments leading to egg fertilization and larval hatch used SPE for the Japanese eel, while CPE was used for the European eel, applying constant and increasing doses, respectively [46,47]. Constant dosages refer to a weekly treatment with PE relative to initial female body weight (BW), e.g. 18.75, 20, or 25 mg kg^-1^ BW [39,48,49], while increasing dosages start at a low dose and increase over time, e.g. 10 mg kg^-1^ BW (week 1–3), 20 mg kg^-1^ BW (week 4–6), 30 mg kg^-1^ BW (week 7–9), and 40 mg kg^-1^ BW (week 10–16) [50,51]. Many studies adopted the constant dose SPE protocol used for Japanese eel [52], including the American eel (*A*. *rostrata*) [53], and the New Zealand longfin eel (*A*. *dieffenbachii*) [54] or used both PE types, as in Australian shortfin eel (*A*. *australis*) [54,55]. In spite common use of these assisted reproduction protocols with either SPE or CPE, there are few comparative studies on their effects [56,57]. Pituitary glands are not standardized products and may vary in the levels of gonadotropins and other pituitary hormones they contain, which may affect follicle development, egg quality, and offspring developmental competence.

Against this background, we aimed to elucidate effects of CPE and SPE on European eel reproductive success and offspring quality. We compared the effects of constant dose treatment schemes on reproductive parameters including egg characteristics, fertilization success, occurrence of cleavage abnormalities, embryonic survival, and hatch success in a standardized experimental design. Gene expression analyses were performed to assess mRNA transcript abundance of genes in ovary, unfertilized eggs, and embryos at regular intervals throughout development.

## Material and methods

### Ethics statements

All fish were handled in accordance with the European Union regulations concerning the protection of experimental animals (Dir 2010/63/EU). Eel experimental protocols were approved by the Animal Experiments Inspectorate (AEI), Danish Ministry of Food, Agriculture and Fisheries (permit number: 2015-15-0201-00696). Each individual fish was anesthetized before tagging, biopsy, and stripping of gametes, and euthanized after stripping (females) or at the end of the experiment (males) using an aqueous solution of ethyl p-aminobenzoate (benzocaine, 20 mg L^-1^, Sigma Aldrich, Germany).

### Fish and experimental design

#### Broodstock establishment

Female silver eels were caught during down-stream migration and matured in two consecutive trials in 2016 and 2017. In 2016, 25 female silver eels (length = 76.18 ± 1.23 cm; weight = 879.16 ± 40.49 g) were caught at Lower Bann, Toomebridge, Northern Ireland, while in 2017, 26 female silver eels (length = 64.42 ± 1.21 cm; weight = 535.33 ± 39.93 g) were caught in Klitmøller Å, Lake Vandet, Denmark. The female eels were transported to the EEL-HATCH experimental facility in Hirtshals, Denmark, using an aerated freshwater tank. On arrival, fish were randomly distributed into replicated 1150 L tanks, connected to two Recirculating Aquaculture Systems (RAS), at a density of 10–15 females per tank, where one RAS unit was allocated for each hormonal treatment. In both years, female eels were equally distributed into the two hormonal treatment groups. Hence, 27 females were allocated to CPE treatment (2016: n = 12; 2017: n = 15) and 24 females to SPE treatment (2016: n = 13; 2017: n = 11).

In both trials, male eels originated from Stensgård Eel Farm, where they were raised from glass eels on a formulated diet (DAN-EX 2848, BioMar A/S, Denmark) at a temperature of ~23°C. In 2016, experiments comprised 60 male eels (length = 38.2 ± 2.1 cm, weight = 105.5 ± 15.3 g), and in 2017, 88 males (length = 38.5 ± 2.1 cm, weight = 114.7 ± 15.8 g). After transport to the facility, males were randomly distributed in four tanks (485 L) connected to a RAS unit at a density of ~15–22 eels per tank.

For acclimatization to oceanic conditions, salinity was gradually increased from 10 to 36 PSU over 14 days using Blue Treasure Aquaculture Salt (Qingdao Sea-Salt Aquarium Technology Co. Ltd. Qingdao, China), while temperature was adjusted from ~16°C to 20°C. Subsequently, each individual was tagged with a passive integrated transponder (PIT tag) in the dorsal muscle, while initial length and weight were recorded. During the experiment, male and female broodstock were maintained at ~20°C and ~36 PSU under 12 h—12 h light regime, with a 30 min twilight in the morning and evening to resemble the Sargasso Sea photoperiod.

#### Assisted reproduction and hormonal treatment

After acclimatization, vitellogenesis in the female broodstock was induced by weekly intramuscular injections of either CPE or SPE, each at 18.75 mg kg^-1^ initial BW for 10–21 weeks [58]. Salmon and carp pituitary extracts were obtained from Argent Chemical Laboratories, Washington, USA, diluted in NaCl 0.9 g/L, ground, and centrifuged at 3600 RPM for 20 minutes, following [52,59], and supernatants stored at -20°C until use. Dependent on body-weight increase and oocyte developmental stage, monitored by biopsies, an additional injection of the respective hormone was given to each female as a primer [49,60]. After 12–24 hours the female received an injection of 17α,20ß-dihydroxy-4-pregnen-3-one (DHP) (Sigma-Aldrich, St. Louis, MO, USA) at 2 mg kg^-1^ current BW to induce follicular maturation and ovulation [59]. Males received weekly injections of human chorionic gonadotropin (Sigma-Aldrich, Missouri, USA) at 150 IU/fish [58]. Prior to spawning, milt from 3–5 males was collected, sperm concentration standardized [61], and the dilution kept in an immobilizing medium [62]. Eggs were strip-spawned and fertilized using a standardized sperm to egg ratio [63,64]. After five min, eggs were transferred to 20 L buckets filled with ~15 L reverse osmosis water salted to ~36 PSU with Blue Treasure (Qingdao Sea-Salt Aquarium Technology Co., Ltd., Qingdao, China) at ~19°C. After 60 min, the floating layer of eggs/embryos was transferred to a second bucket (as above) and kept for 60 min. Eggs/embryos were taken from the floating layer of the separation bucket and subsequently incubated in 10 × 1 L glass beakers (~5000 eggs/embryos per L) filled with filtered UV-treated seawater (FUV seawater; filter size: 10, 5, 1 μm) and supplemented with rifampicin and ampicillin (each 50 mg L^-1^, Sigma-Aldrich, Missouri, USA). Subsequent rearing occurred in a temperature incubator at 18°C [65] and 36 PSU. Additionally, 6 × 200 mL sterile tissue culture flasks filled with FUV seawater and supplemented with rifampicin and ampicillin (each 50 mg L^-1^, Sigma-Aldrich, Missouri, USA) were stocked with eggs/embryos and incubated as above. 3 flasks stocked with ~2500 eggs/embryos were used to follow embryonic development and 3 flasks stocked with ~600 eggs/embryos were used to analyze hatch success.

#### Data collection and image analyses

For each female, initial length and weight, weekly weights and weight at DHP injection were recorded as well as the number of weeks until spawning and the time between priming and DHP were recorded. The weight of stripped eggs (% initial weight) was recorded prior to fertilization and unfertilized eggs were sampled for dry weight (3 × 0.1 mL) and gene expression (see below). For dry weight, samples were kept in the oven at 60°C for 24 h and weighed. At 0.5 hpf, the amount of floating eggs (%) was determined in a 25 mL volumetric column. Digital images were used throughout the experiment to document oocyte, egg, and embryonic development. Here, all images were taken with a Nikon Eclipse 55i microscope equipped with a Nikon digital sight DS-Fi1 Camera, while analyses used NIS Elements image software (Nikon Corporation, Tokyo, Japan). A digital image of the ovarian biopsy was taken, when females were primed or DHP was given, and oil droplet size was measured subsequently. Ten of the largest oil droplets from ten oocytes at average stage were measured per female. In order to calculate fertilization success and embryonic survival, sub-samples from the embryonic development flasks were taken at 2, 3, 4, 5, 6, 7, 8, 16, 24, 32, 40, and 48 hpf and digital images were obtained. Fertilization success was measured from the digital images taken at 4 hpf, where eggs were categorized as fertilized when > 4 blastomeres could be observed and fertilization success was calculated as the percentage of fertilized divided by the total number eggs (obtained from the floating layer) [63]. At the remaining sampling points, embryonic survival was then calculated by counting the number of dead and alive eggs and expressing it as a percentage. Additionally, morphological measurements were conducted at 4 hpf, where total egg area, yolk area, and oil droplet area was measured. Cleavage abnormalities were also determined at 4 hpf by counting the number of eggs with regular and abnormal cell cleavages. Hatch success from flasks was obtained by counting hatched larvae and dead eggs and was expressed as the number of hatched larvae divided by the total number of floating eggs.

### Gene expression

Samples for gene expression were taken from the ovarian tissue of the female after spawning, and from the unfertilized eggs and embryos at 2, 4, 8, 24, 32, and 48 hpf. The samples were stored in Eppendorf vials filled with RNA later, then kept in the fridge at 4°C for 24 hours and subsequently at -20°C until analysis. RNA was extracted using the NucleoSpin RNA kit (Macherey-Nagel, Germany) according to manufacturer’s instructions. RNA concentration and purity was analyzed by spectrophotometry using Nanodrop One (Thermo Fisher Scientific, USA). From the resulting total RNA, 1 μg was transcribed using qScript cDNA Synthesis Kit (Quantobio, Germany), following the manufacturer’s instructions. This included a step to permanently inactivate all trace levels of DNase activity using PerfeCta DNase I (RNase free) (Quantabio, Germany). Primers of eight genes (*thαa*, *foxr1*, *npm2*, *zar1*, *phb2*, *c3*, *igm*, *il1β*) were retrieved from previous studies (Table 1). Primers of four genes (*ccna2*, *ccna1*, *ccnb1*, *ccnb2*) were designed on the basis of the coding sequences of the closely related species *A*. *japonica*, publicly available in Genebank, National Center for Biotechnology Information (NCBI). Primers for 12 genes (*cei*, *igfr-1b*, *cea*, *cdhr2*, *cldnd*, *dcbld1*, *epcam*, *dicer1*, *oct4*, *sox2*, *neurod4*, *neurog1*) were designed based on the most accurate assembly and annotation of the European Eel genome [66] available at https://dataverse.no/dataset.xhtml?persistentId=doi:10.18710/L7GO8T. Here, gene prediction and exon coordinates were determined by the authors with Augustus v 2.4 [67], and annotation was performed using Blast2GO v 2.4.8 [68]. The coding sequences used for our analyses were then submitted to Genebank, NCBI and the related accession numbers are given in Table 1. Primers were designed using primer 3 software v 0.4.01. All primers and predicted amplicons were tested in silico for specificity using blast (https://blast.ncbi.nlm.nih.gov/Blast.cgi). Subsequently, from all samples, the expression of the 24 genes was analyzed with two technical replicates, using the qPCR BiomarkTM HD system (Fluidigm) based on 96.96 dynamic arrays (GE plates), as previously described [69]. In brief, a pre-amplification step was conducted with a 500 nM pool of all primers in PreAmp Master Mix (Fluidigm) and 1.25 μL cDNA per sample run in a thermocycler for 2 min at 95°C; 10 cycles: 15 s each at 95°C and 4 min at 60°C. Obtained PCR products were then diluted 1:5 with low EDTA-TE buffer. The preamplified product was loaded onto the chip with SsoFast-EvaGreen Supermix Low Rox (Bio-Rad) and DNA-Binding Dye Sample Loading Reagent (Fluidigm). Primers were loaded onto the chip at a concentration of 50 μM in Assay Loading Reagent (Fluidigm) and low EDTA-TE Buffer. The chip was run according to the Fluidigm 96.96 PCR protocol with a Tm of 60°C. qBase + software verified stability of housekeeping gene expression throughout analyzed samples (M < 0.4; according to [70]). Gene expression was normalized (ΔCt) to the geometric mean of the four most stable reference genes (*ccna2*, *cei*, *thαa*, *igfr-1b*). Further analysis of gene expression was carried out according to the 2^-ΔΔCt^ method, in relation to a random unfertilized egg sample, according to [71].

**Table 1:** Sequences of European eel, *Anguilla anguilla* primers used for amplification of genes by qRT-PCR. Full name and abbreviation is given for each gene with function, accession numbers and references for primers retrieved from previous studies. Primers were designed based on the coding sequence (cds), that have been retrieved based on the predicted annotation of the European eel reference genome [66], complemented with cds of *A*. *japonica*.

Abbreviation	Full name	Function	Primer Sequence (5’ 3’) (F: Forward; R: Reverse)	Reference/Accession
*ccna2*	Cyclin A2	Reference	F: ATGGAGATAAAATGCAGGCCT	AB061443.1
			R: AGCTTGCCTCTCAGAACAGA	
*cei*	Cellular island	Reference	F: CCTCAAACACCCCAACATCC	MT531390
			R: AGCTCCTCCATGTACGTTGC	
*thαa*	Thyroid hormone receptor *αa*	Reference	F: GCAGTTCAACCTGGACGACT	Politis et al., [72]
			R: CCTGGCACTTCTCGATCTTC	
*igfr-1b*	Insulin like growth factor receptor 1b	Reference	F: ATGGGAATCTTCAGCTCTTTAGA	MT531391
			R: TCAAACTCCTCCTCCAAGCT	
*foxr1*	Forkhead box R1	Cell division	F: CCTCGTCCAGCGAATATCTTCTT	Geffroy et al., [73]
			R: TGTTTTGAGCGAGATTCAGCTTC	
*cea*	Cellular atoll	Cell division	F: AGCACTCTGTCGAAGGAAGT	MT531392
			R: ACCTTGATCTTCCCCACCAG	
*ccna1*	Cyclin A1	Cell cycle control	F: ACCTGCTTCTCAAGGTCCTC	AB061442.1
			R: CCTTGGACGGAACATGTAGC	
*ccnb1*	Cyclin B1	Cell cycle control	F: TCAACCTCAAGCTGACGGAG	AB183431.1
			R: CTGCATCTCCCACACCCAT	
*ccnb2*	Cyclin B2	Cell cycle control	F: GTGTTGCATGATGGGCTTGA	AB183432.1
			R: TGATGCAGAGAAACACACGC	
*npm2*	Nucleoplasmin 2	Cell cycle control	F: AAAGTTGACCGTTGGACCAG	Rozenfeld et al., [20]
			R: GGCCTATGTGAGGCAGTCAT	
*cdhr2*	cadherin-related family member 2 or protocadherin-24	Cell adhesion	F: GTTCCTTCGGTCACCACAAC	MT531393
			R: TGTGTGACCAGGTGCAAATG	
*cldnd*	Claudin d	Cell adhesion	F: CTCCCCAGCCAATGAACAAC	MT531394
			R: ATTCTGTTGTCGGTTGCTGG	
*dcbld1*	Discoidin, CUB and LCCL domain containing 1	Cell adhesion	F: ACCAGTCCACAGAGTTCACC	MT531395
			R: CGTGTGCAGGTAGTCGTAGT	
*epcam*	Epithelial cell adhesion molecule	Cell adhesion	F: TCTTCAGGTCTCTCTCGATGT	MT531396
			R: GCTGGTGAAGGAATATACTCTGG	
*zar1*	Zygote arrest 1	Oocyte-embryo transition	F: TGAGGTTTCAGTTCTTGGAGCAG	Geffroy et al., [73]
			R: TAAACCTTGTTGGTTCCCTGGAC	
*dicer1*	Dicer1	microRNA regulation	F: CGGTCGTCTTAAACAGGCTTATA	MT531397
			R: ACCTCCTCCTGTTTGCGAAA	
*oct4* (also known as *pou5f1* or *pou2*)	Octamer-binding transcription factor 4	Pluripotency regulation/ MZT activation	F: AACAGTTTGCCAAGGAGCTG	MT531398
			R: GCACATGTTCTTAAAGCTCAGC	
*sox2*	Sox2	Pluripotency regulation/ MZT activation	F: GTCCTTTCATCGACGAAGCG	MT531399
			R: TGATTTACTCCCGCACCCAA	
*phb2*	Prohibitin 2	cell signaling	F: AAATGTTGGGAGAGGCTGTG	Rozenfeld et al., [20]
			R: ACCGTCTTGGCGATATTCTG	
*neurod4*	Neuronal differentiation 4	Neurogenesis	F: TTCCTGTCCTCGCACCAGTA	MT531400
			R: AAGGAGTCGAAGGCCATGTC	
*neurog1*	Neurogenin 1	Neurogenesis	F: CAGGATGCACAACCTCAATG	MT531401
			R: TGCAATTCGGATTGTCTCTG	
*c3*	Complement component c3	Immune response	F: AATATGTGCTCCCAGCCTTC	Miest et al., [74]
			R: GATAACTTGCCGTGATGTCG	
*igm*	Immunoglobulin M	Immune response	F: CCAAGGACCATTCTTTCGTC	Miest et al., [74]
			R: ACTGGCTTTCAGGAAGATGC	
*il1β*	Interleukin 1β	Immune response	F: ATTGGCTGGACTTGTGTTCC	Miest et al., [74]
			R: CATGTGCATTAAAGCTGACCTG	

### Statistical analyses

Data were analyzed using SAS Statistical Software (version 9.4; SAS Institute Inc., Cary, North Carolina). Prior to analysis, residuals were tested for normality (Shapiro–Wilk test) and homogeneity of variances (plot of residuals vs. fitted values). Data deviating from normality or homoscedasticity were log_10_ or arcsine square-root-transformed. Alpha was set at 0.05. Tukey analysis was used to compare least-squares means between treatments. Akaike (AIC) and Bayesian (BIC) information criteria were used to assess which covariance structure fitted the data most appropriately [75]. The effects of hormonal treatment on embryonic survival throughout development (2, 3, 4, 5, 6, 7, 8, 16, 24, 32, 40, 48 hpf) and on gene expression throughout development (unfertilized egg, 2, 4, 8, 24, 32, 49 hpf) were tested using a series of repeated measures mixed-effect model ANOVAs. Female ID (individual females and their offspring) was considered random in all models. No significant interactions were detected for any of the tested dependent variables and all models were re-run with the interaction effects removed, analyzing main effects separately [76]. The effects of hormonal treatment on initial length, initial weight, time until spawning, oil droplet stage at priming and at DHP stage, weight increase of females, stripped eggs, floating eggs, dry-weight of unfertilized eggs, fertilization success, cleavage abnormalities and hatch success were tested using student t-tests. Linear and quadratic regression functions were used to analyze the relationship between cleavage abnormalities at 4 hpf and embryonic survival at 48 hpf, as well as relationships between gene expression in the ovary and unfertilized eggs and between gene expression and offspring quality parameters. In cases where both regression functions were significant, F-statistics were used to evaluate best fit.

For female and quality parameters, ANOVA models were first run testing the effect of broodstock series (2016, 2017), hormonal treatment and their interaction. While initial length (p < 0.0001) and weight (p < 0.0001) differed between female broodstock from the two locations, quality parameters were similar between female broodstocks, including fertilization success (p = 0.7832), cleavage abnormalities (p = 0.6711), embryonic survival (p = 0.4538), and hatch success (p = 0.6258). Data from the two experiments were therefore pooled. Comparing the two resulting treatment groups, no significant difference was found in the initial weight between CPE (686. 3 ± 49.7 g) and SPE females (723.5 ± 55.9 g; p = 0.620). Likewise, the initial length between females from CPE (70.1 ± 1.6 cm) and SPE (70.3 ± 1.8 cm) treatment did not differ (p = 0.952).

## Results

### Reproductive success and offspring development

Overall, 51 females were included in the combined analyses of the two trials. Female information and reproductive success for each treatment are shown in Table 2. The female weight increase (% IW) until DHP stage was similar for the two treatments (p = 0.161). The number of weeks until spawning (p = 0.189) and the time between primer and DHP (p = 0.659) also did not differ between hormonal treatments. There was no difference in the oil droplet size at priming stage (p = 0.142), or at DHP stage (p = 0.139) between hormonal treatments. The overall percentage of females that responded to the treatment and were stripped was higher in the CPE treatment, while the percentage of stripped females producing fertilized eggs and hatched larvae was higher in the SPE treatment (Table 2).

**Table 2:** Data on females and reproductive success of European eel, *Anguilla anguilla* in two successive trials. Different lower-case letters represent a significant statistical difference (p < 0.05).

Parameter	CPE	SPE
n females in trials	27	24
Time until spawning (wks)	14.19±3.93 ^a^	15.58±2.61 ^a^
Time between primer and DHP (h)	20.38±0.75 ^a^	21.24±1.05 ^a^
Oil droplet diameter at priming stage (μm)	109.9±33.68 ^a^	95.33±26.94 ^a^
Oil droplet diameter at DHP stage (μm)	157.4±31.9 ^a^	145.1±16.84 ^a^
Stripped females (%)	96.3 (n = 26)	79.2 (n = 19)
Stripped females with fertilized eggs (%)	57.7 (n = 15)	89.5 (n = 17)
Stripped females with hatched larvae (%)	50.0 (n = 13)	73.7 (n = 14)
Weight increase (% IW)	22.9±1.9 ^a^	26.0±0.9 ^a^
Stripped eggs (% IW)	42.4±1.9 ^a^	42.1±2.4 ^a^
Floating eggs (%)	55.3±8.8 ^a^	79.7±6.1 ^b^
Dry-weight unfertilized egg (mg egg^-1^)	0.060±0.002 ^a^	0.061±0.001 ^a^
Egg size (mm^2^)	1.58±0.09 ^a^	1.68±0.09 ^a^
Fertilization success (%)	53.37±4.95 ^a^	54.75±6.56 ^a^

**Fig 1:**
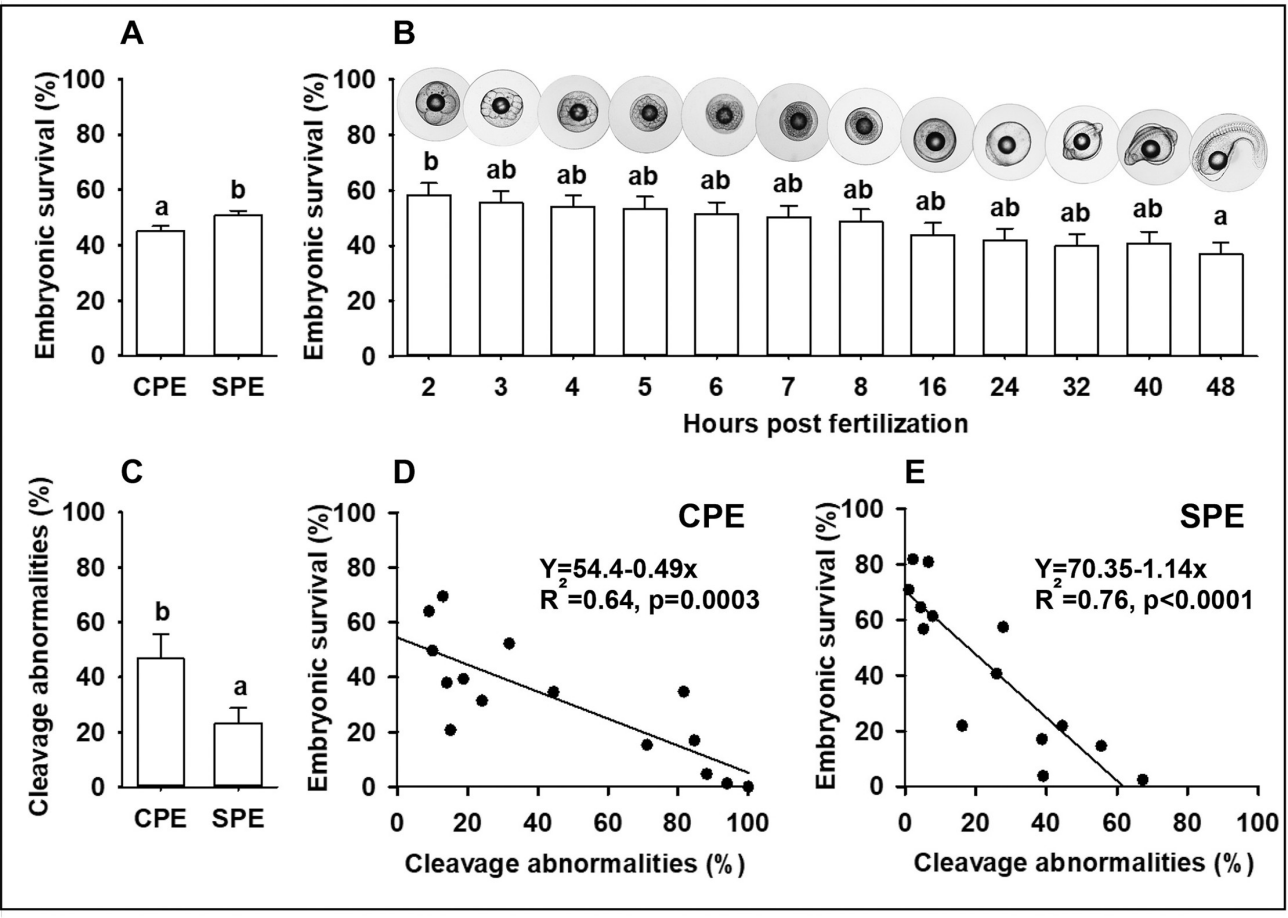
Embryonic survival and cleavage abnormalities in embryos from hormone-treated European eel, *Anguilla anguilla*. Embryonic survival in relation to (A) hormonal treatment and (B) age in hours post fertilization, (C) difference in proportions of cleavage abnormalities at 4 hpf among hormonal treatments and relationships between cleavage abnormalities at 4 hpf and embryonic survival at 48 hpf for (D) CPE and (E) SPE treatment. Values for bar plots represent means (± SEM) of embryos at each age and treatment. Different lower-case letters represent a significant statistical difference (p < 0.05).

**Fig 2:**
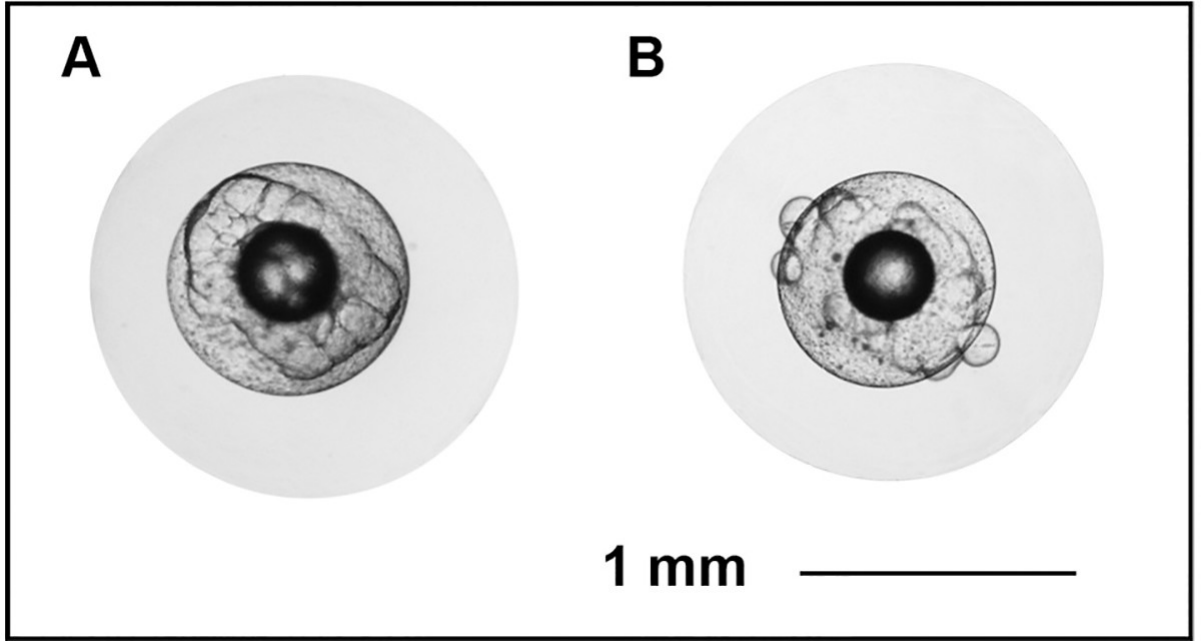
Examples of normal development and cleavage abnormalities in European eel, *Anguilla anguilla* embryos. Eggs with (A) normal development and (B) occurrence of cleavage abnormalities at 4 hours post fertilization. Scale bar represents 1 mm.

The number of stripped eggs relative to body weight (p = 0.926) and the dry-weight of unfertilized eggs (p = 0.865) did not differ between CPE and SPE females. The percentage of floating eggs was higher in females from the SPE treatment than in females from the CPE treatment (p = 0.038), but the fertilization success of eggs from the floating layer (p = 0.868) and egg size at 4 hpf (p = 0.428) did not differ. Embryonic survival was higher for SPE treated females than CPE (p = 0.029; Fig 1A), while embryonic survival in general decreased over time (p = 0.003; Fig 1B). Reduced embryonic survival coincided with higher proportions of cleavage abnormalities at 4 hpf in embryos obtained from CPE treated females (p = 0.037; Fig 1C). The relationship between cleavage abnormalities and embryonic survival at 48 hpf was significant for both treatments (Fig 1D and 1E). Examples of normal egg development and occurrence of cleavage abnormalities are shown in Fig 2.

The number of females with viable offspring decreased over time, with about half of the CPE and three quarters of the SPE treated females succeeding in larval production (Table 2). The resulting hatch success was on average higher for SPE (37.87 ± 7.25%) than for CPE (26.54 ± 6.19%) treated females, but the difference between treatments was not significant (p = 0.245).

### mRNA transcript abundance and gene expression patterns

Detailed results of gene expression analyses are shown in Table 3. The relationship between expression levels in the ovary and the unfertilized egg for SPE was significant for 13 genes (*cdhr2*, *dcbld1*, *epcam*, *foxr1*, *cea*, *ccna1*, *ccnb1*, *oct4*, *sox2*, *neurod4*, *c3*, *igm*, *il1β*). For CPE, the relationship was significant for 7 genes (*cdhr2*, *epcam*, *ccna1*, *oct4*, *neurog1*, *c3*, *igm*) with six of these overlapping between treatments. For all genes, the relationship was best described by a positive linear regression.

**Table 3:** Gene expression in ovary, unfertilized eggs, and embryos in two successive trials with hormone-treated European eel, *Anguilla anguilla*. Best fitting relationship between expression of the gene in the ovary of female and levels of mRNA transcripts in unfertilized egg for each treatment and mRNA abundance from unfertilized egg to embryos (shortly before hatch) related to hormonal treatment (CPE and SPE) and age of embryos (in hpf).

Gene	Relationship ovary–unfertilized eggs—CPE			Relationship ovary–unfertilized eggs—SPE			Model on unfertilized eggs and embryos			
	Equation	R^2^	p-value	Equation	R^2^	p-value	CPE (mean ± SE)	SPE (mean ± SE)	Hormonal treatment (p-value)	Age (p-value)
*cdhr2*	Y = 0.01+0.67x	0.951	**<0.0001**	Y = -0.52+0.72x	0.954	**<0.0001**	9.31±0.89	6.18±0.88	**0.014**	0.208
*cldnd*	Y = 1.38–0.08x	0.003	0.847	Y = 0.36+0.43x	0.086	0.290	0.54±0.04	0.41±0.08	**0.028**	**<0.0001**
*dcbld1*	Y = 1.58–0.58x	0.195	0.076	Y = -5.03+0.58x	0.797	**<0.0001**	3.33±21.71	99.13±21.36	**0.0003**	0.958
*dicer1*	Y = 1.25+0.32x	0.023	0.534	Y = 1.03+0.25x	0.092	0.273	1.23±0.03	1.26±0.03	0.354	**<0.0001**
*epcam*	Y = 1.26+0.17x	0.637	**0.0001**	Y = 1.04+0.16x	0.906	**<0.0001**	5.88±0.70	8.85±1.63	**0.012**	**0.0003**
*foxr1*	Y = 0.72+0.35x	0.084	0.243	Y = 0.74+0.32x	0.318	**0.029**	0.67±0.05	0.48±0.11	**0.013**	**<0.0001**
*cea*	Y = 0.30+0.64x	0.170	0.089	Y = 0.11+0.83x	0.740	**<0.0001**	0.74±0.03	0.67±0.07	0.156	**<0.0001**
*ccna1*	Y = 1.20+1.04x	0.239	**0.047**	Y = 1.10+0.82x	0.692	**0.0001**	1.34±0.07	1.33±0.06	0.852	**<0.0001**
*ccnb1*	Y = 3.37–1.76x	0.076	0.269	Y = 0.55+0.90x	0.335	**0.030**	1.16±0.02	1.06±0.03	**0.013**	**<0.0001**
*ccnb2*	Y = 2.67–1.35x	0.065	0.306	Y = 1.02+0.16x	0.012	0.702	0.96±0.05	1.04±0.12	0.366	**<0.0001**
*npm2*	Y = 2.97–0.45x	0.057	0.342	Y = 0.88+0.15x	0.116	0.215	1.04±0.08	1.05±0.15	0.97	**<0.0001**
*phb2*	Y = 1.43–0.02x	0.007	0.743	Y = 0.93+0.05x	0.032	0.525	5.31±0.40	5.90±0.86	0.393	**<0.0001**
*oct4*	Y = -0.17+1.55x	0.336	**0.009**	Y = 0.29+0.90x	0.567	**0.001**	0.80±0.03	0.92±0.08	**0.048**	**<0.0001**
*sox2*	Y = 0.86+0.15x	0.167	0.103	Y = -0.16+0.55x	0.996	**<0.0001**	1070.99±89.07	1137.89±112.47	0.708	**<0.0001**
*zar1*	Y = 3.60–0.11x	0.035	0.460	Y = 1.12+0.009x	0.016	0.649	0.56±0.06	0.47±0.13	0.364	**<0.0001**
*neurod4*	Y = 1.82–0.47x	0.079	0.242	Y = 0.27+0.21x	0.878	**<0.0001**	1299.55±115.67	1279.14±217.08	0.378	**<0.0001**
*neurog1*	Y = 1.37+0.63x	0.376	**0.007**	Y = 1.31+0.14x	0.244	0.061	238.84±12.57	247.10±13.76	0.245	**<0.0001**
*c3*	Y = 0.57+0.21x	0.231	**0.037**	Y = 0.09+0.40x	0.646	**0.0003**	1150.66±125.19	829.36±285.06	0.314	**<0.0001**
*igm*	Y = 0.32+0.15x	0.271	**0.022**	Y = 0.03+0.40x	0.699	**0.0001**	0.53±0.06	0.85±0.14	**0.002**	**<0.0001**
*il1β*	Y = 2.03+0.59x	0.012	0.651	Y = 0.26+0.28x	0.282	**0.041**	0.75±0.06	0.78±0.06	0.768	**<0.0001**

**Fig 3:**
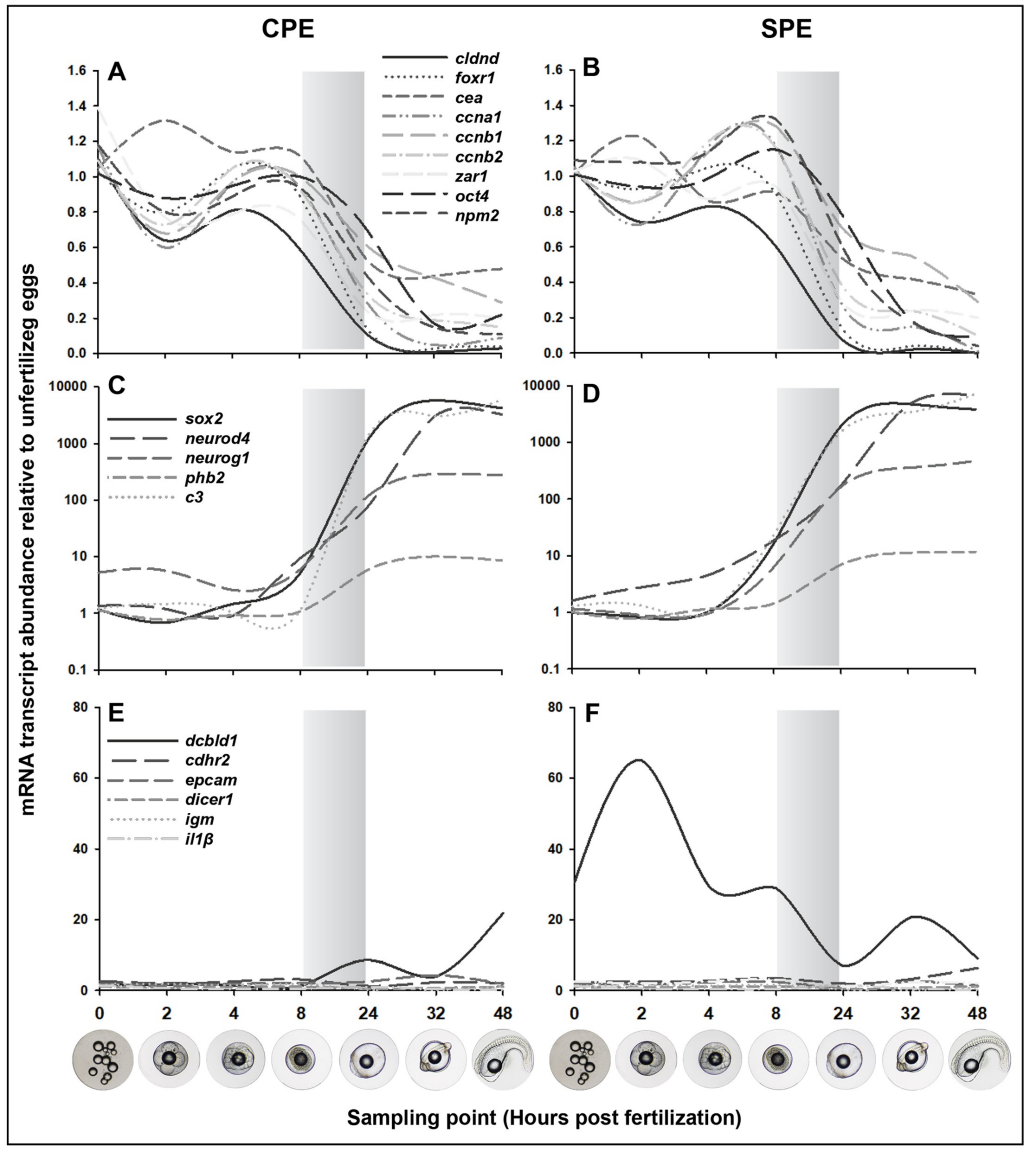
mRNA transcript abundance in unfertilized eggs and developing embryos of hormone-treated European eel, *Anguilla anguilla*. Conceptual overview–Expression (2^-ΔΔCt^) was calculated in relation to the average abundance in the unfertilized eggs of each gene. Relative abundance of *cldnd*, *foxr1*, *cea*, *ccna1*, *ccnb1*, *ccnb2*, *zar1*, *oct4*, *npm2* for (A) CPE and (B) SPE treatment. Relative abundance of *sox2*, *neurod4*, *neurog1*, *phb2*, *c3* for (C) CPE and (D) SPE treatment. Relative abundance for *cdhr2*, *dcbld1*, *epcam*, and *dicer1*, *igm*, *il1β* for (E) CPE and (F) SPE treatment. Bars represent timeframe of maternal-to-zygotic transition (MZT).

The statistical model for analysis of hormonal treatment and age on mRNA abundance from unfertilized eggs throughout embryogenesis showed that the transcript levels of eight genes differed significantly (Table 3). *dcbld1*, *epcam*, *oct4*, and *igm*, which are associated with cell adhesion, MZT activation, and immune response, showed significantly higher mRNA levels in offspring from SPE than from CPE treated females. In contrast, *cdhr2*, *cldnd*, *foxr1*, and *ccnb1*, associated with cell division, cell cycle control, and cell adhesion, showed significantly higher mRNA levels in offspring from CPE than from SPE treated females. Significant variations in patterns of mRNA abundance throughout embryonic development were observed for all genes, with the exception of *cdhr2* and *dcbld1*. Here, *cldnd*, *foxr1*, *cea*, *ccna1*, *ccnb1*, *ccnb2*, *zar1*, *oct4*, and *npm2* showed a similar pattern with relatively stable abundance during the first eight hours until MZT (Fig 3A and 3B). After this, mRNA levels for these genes declined greatly, remaining low until hatch. In contrast, mRNA levels of *sox2*, *neurod4*, *neurog1*, *phb2*, and *c3* were low in the unfertilized eggs and first hours post fertilization, but increased steeply during MZT and remained high thereafter (Fig 3C and 3D). Up to almost 100,000-fold differences were detected in the expression during later embryonic development. Lastly, levels of mRNA transcripts were low for *epcam*, *dicer1*, *igm*, *il1β*, while a deviating pattern with large differences between treatments was observed for *dcbld1* during embryonic development (Fig 3E and 3F).

### mRNA abundance and developmental competence

The relationships between relative mRNA levels of analyzed genes and offspring quality parameters are shown in Table 4. Significant relationships between relative mRNA levels at 2 hpf and the occurrence of cleavage abnormalities (at 4 hpf) were found for three genes. For *dicer1*, no relationship was found for CPE females, however, for SPE females the relationship was best explained by a negative linear regression. The relationship between *epcam* and cleavage abnormalities for CPE females was best explained by a negative linear regression, while no relationship was found for the SPE treatment. The relationship for *zar1* was best explained by a negative linear regression for SPE females, but was not significant for CPE females.

**Table 4:** Relationship between mRNA abundance of specific genes in eggs and embryos at selected sampling points and offspring quality parameters for the two treatments (CPE and SPE) in European eel, *Anguilla anguilla*, including best fitting equation and significance levels for genes, where a significant effect was found for at least one of the treatments.

				CPE			SPE		
Gene	Function	Relative abundance	Quality parameter	Equation	R^2^	p-value	Equation	R^2^	p-value
*dicer1*	microRNA regulation	2 hpf	Cleavage abnormalities	Y = 52.67–5.57x	0.002	0.876	Y = 94.24–60.74x	0.338	**0.023**
*epcam*	cell adhesion	2 hpf	Cleavage abnormalities	Y = 67.28–9.22x	0.357	**0.024**	Y = 24.95–0.30x	0.004	0.831
*zar1*	MZT	2 hpf	Cleavage abnormalities	Y = 76.42–31.82x	0.149	0.155	Y = 62.07–35.30x	0.324	**0.034**
*zar1*	MZT	8 hpf	Survival (48 hpf)	Y = -7.26+44.41x	0.326	**0.042**	Y = -15.3+47.07x	0.350	**0.020**
*sox2*	MZT	8 hpf	Survival (48 hpf)	Y = 15.83+3.61x	0.729	**0.0002**	Y = 7.89+17.17x	0.697	**0.0004**
*foxr1*	cell division	8 hpf	Survival (48 hpf)	Y = -23.32+45.92x	0.265	0.072	Y = -56.24+75.42x	0.495	**0.003**
*cldnd*	cell adhesion	8 hpf	Survival (48 hpf)	Y = 0.75+41.59x	0.104	0.282	Y = -68.68+134.63x	0.364	**0.017**
*phb2*	normal mitochondrial function	8 hpf	Survival (48 hpf)	Y = -24.82+44.64x	0.204	0.122	Y = -32.78+49.99x	0.269	**0.047**
*neurod4*	Neurogenesis	8 hpf	Survival (48 hpf)	Y = 20.60+0.96x	0.419	**0.017**	Y = 12.89+3.21x-0.04x^2^	0.516	**0.013**
*neurog1*	Neurogenesis	8 hpf	Survival (48 hpf)	Y = 11.43+1.56x	0.672	**0.0006**	Y = 13.35+4.16x-0.05x^2^	0.509	**0.014**
*Ccna1*	cell cycle control	32 hpf	Hatch success	Y = 5.70+379.13x-478.20x^2^	0.524	**0.024**	Y = 21.41+88.96x	0.408	**0.025**
*npm2*	nuclear organization	32 hpf	Hatch success	Y = 32.02–19.88x	0.040	0.509	Y = 11.19+151.72x	0.473	**0.013**
*oct4*	MZT	32 hpf	Hatch success	Y = -5.12+545.65x-513.65x^2^	0.498	**0.032**	Y = 21.11+115.98x	0.357	**0.040**
*neurod4*	Neurogenesis	32 hpf	Hatch success	Y = 69.93–0.009x	0.048	0.473	Y = 82.06–0.03x+0.01x^2^	0.605	**0.015**
*neurog1*	Neurogenesis	32 hpf	Hatch success	Y = 25.03+0.004x	0.00	0.931	Y = 93.25–0.09x	0.601	**0.003**

For seven genes, the relationship between the relative mRNA levels at 8 hpf and embryonic survival at 48 hpf was significant for at least one of the treatments. For *zar1*, the relationships were best explained by positive linear regressions for CPE as well as SPE. The relationship between *sox2* and survival was also best explained by positive linear regressions for both treatments. No significant relationship was found between the mRNA levels of *foxr1* and survival for CPE females, while a significant positive linear regression was found for SPE females. The relationship between the mRNA levels of *cldnd* and survival was also non-significant for CPE females but best explained by a positive linear regression for the SPE treatment. Similarly, no significant relationship was found for CPE females between *phb2* levels and survival, while the relationship for SPE females was best explained by a positive linear regression. For *neurod4*, a significant relationship was found for both treatments. Here, the relationship for CPE females was best explained by a positive linear regression, whereas for SPE females it was a positive quadratic regression. Similar results were found for *neurog1*, where the relationship for CPE females was best explained by a positive linear and for SPE females by a quadratic regression.

Lastly, five genes showed a significant relationship between the mRNA abundance at 32 hpf and hatch success. The relationship between the mRNA abundance of *ccna1* and hatch success was best explained by a positive quadratic regression for CPE and a positive linear regression for SPE females. For *npm2*, no relationship was found for CPE females, while the relationship was best explained by a positive linear regression for SPE females. For *oct4*, significant relationships were found for both treatments and best explained by a positive quadratic regression for CPE and a linear regression for SPE females. No significant relationship was found between the abundance of *neurod4* and hatch success for CPE females, however, for SPE females, the relationship was best explained by a negative quadratic regression. Similarly, no relationship was found between the abundance of *neurog1* and hatch success for CPE females. Here, the relationship for SPE females was best explained by a negative linear regression.

## Discussion

Carp and salmon pituitary extracts administered using identical preparation protocols and treatment schemes caused differences in female responsiveness, egg quality, and embryonic developmental competence in European eel. Differences in embryonic survival were associated with the abundance of mRNA transcripts of genes involved in zygotic formation and embryogenesis. Among the 20 examined genes, several were associated with occurrence of cleavage abnormalities, embryonic survival, and/or hatch success.

### Induced vitellogenesis and egg quality

Assisted reproduction techniques are commonly used in aquaculture to enhance reproductive success including a variety of hormonal treatments depending on the targeted stage of the reproductive cycle [6]. Resulting differences in response to hormonal therapy may be particularly great in anguillid eels due to dopaminergic inhibition [37,58] which requires repeated treatment, most commonly weekly, for a prolonged period, to sustain gametogenesis and reach gamete maturation. In the present study, both CPE and SPE induced vitellogenesis and led to production of viable larvae, however with differences in responsiveness and embryonic survival. More CPE treated females responded and reached the follicular maturation stage, while the proportion of SPE treated females that produced viable eggs and embryos was higher. Such discrepancies in treatment effects could be caused by differences in content and composition of gonadotropins in the pituitary glands or species-specific affinity of eel receptors to carp or salmon gonadotropins as well as other pituitary hormones. Commercially available pituitaries glands are harvested from different fish species close to spawning. While, the exact timing of harvesting will cause variability in species-specific gonadotropin content and composition of pituitary gland, influences of species’ reproductive strategies may be significant. For example, in species, which show synchronous development of oocytes, e.g. salmonids, FSH gene expression is predominant during the early stages of vitellogenesis, whereas LH gene expression becomes elevated as the oocytes approach follicular maturation. In contrast, species, which show asynchronous development of oocytes, e.g. common carp (*Cyprinus carpio*), grass carp (*Ctenopharyngodon idella*), Atlantic halibut (*Hippoglossus hippoglossus*), and gourami (Osphronemidae), the expression of genes for both FSH and LH increases with the progression of ovarian development and peaks during the spawning season [77–80]. It is notable that SPE is derived from Pacific salmon (*Oncorhynchus keta*), a semelparous species with synchronous oocyte development, while CPE is derived from common carp, an iteroparous species with asynchronous oocyte development. In the case of anguillid eels, evidence is accumulating that they are batch spawners with asynchronous or group synchronous oocyte development [41,60,81]. In Japanese eel, sequential expression of FSH and LH at the brain pituitary level has been documented throughout induced ovarian development [82,83] and a similar pattern was also found in European eel with increasing levels of LH and decreasing FSH levels throughout experimental maturation [84]. Such species dependent differences in gonadotropin content and composition may have influenced female responsiveness and ovulation success in the present study as well as follicle development, thereby affecting egg quality.

During reproductive development, mRNA transcripts as well as nutrients (e.g. yolk and vitamins) are incorporated into the growing oocytes [7]. Maternal mRNAs and proteins, which are loaded into the cytoplasm during oocyte development, are instrumental in implementing basic biosynthetic processes during early embryogenesis, with subsequent clearance during MZT [9,12]. We found significant differences between SPE and CPE treatments in mRNA abundance in eggs and embryos for genes related to cell division, cell cycle control, cell adhesion, MZT activation, neurogenesis, and immune response. Observed differences in abundance of several mRNA transcripts during embryogenesis may explain the higher proportion of floating eggs, higher embryonic survival, and lower frequency of cleavage abnormalities in offspring from SPE treated females. High occurrence of cleavage abnormalities led to high embryonic mortality in this study, which has also been found in various species, such as Atlantic cod (*Gadus morhua*) [85], yellowtail flounder (*Limanda ferruginea*) [86], turbot (*Scophthalmus maximus*) [87]. The observed lack of cell adhesion in this study was connected with differences in gene expression patterns.

### Molecular ontogeny and embryonic development

#### Cell adhesion

Abundance of mRNA transcripts of two genes related to cell adhesion, *dcbld1* and *epcam* differed between treatments, with higher mRNA abundances in embryos of SPE treated females. Although expression of *dcbld1* has been associated with cell adhesion during embryonic development, insights into its role are still limited [88]. Rise et al. [25] found high variations in *dcbld1* transcript levels in Atlantic cod eggs with larger than 100-fold differences among females. However, when all females were included in the analysis, no relationship was found between *dcbld1* abundance and egg quality. In our study, *dcbld1* transcript abundance also varied considerably among offspring from individual females, but differed between treatments. The relation between transcript abundance in unfertilized eggs and in the ovary indicated a maternal effect, which in combination with higher mRNA abundance in SPE compared to CPE females may relate to the incomplete cell adhesion observed, compromising the development in European eel embryos.

The second gene, *epcam* has been associated with cell adhesion, migration, proliferation, differentiation and signaling [89–91] with high transcript abundance found in early stages of Atlantic cod [92] and zebrafish [93]. Thus, in zebrafish, expression of *epcam* is required for epithelial morphogenesis during epiboly [89] as well as cell migration in the lateral line system [94]. In our study, high mRNA abundance signaled maternal transfer of *epcam* to eggs and embryos with a positive effect of high transcript levels on performance of embryos obtained from SPE treated females. A negative relationship with cleavage abnormalities was found for CPE. These results indicate that high mRNA abundance of this gene is also important for normal development in European eel embryos.

#### MZT activation

Studies of the expression patterns of *oct4* in medaka (*Oryzias latipes*)[95–98], Nile tilapia (*Oreochromis niloticus*) [99,100], and zebrafish [101–105] have documented its importance for normal fish embryonic development and survival. In zebrafish, maternally inherited *oct4*, *sox2* (*soxb1*), and *nanog* transcripts are responsible for activating zygotic expression and initiating the clearance of maternal mRNA through activation of microRNA, hence they are considered fundamental for successful MZT [103]. We found high mRNA abundance of *oct4* in ovaries, eggs, and embryos, with highest abundance in the SPE treated group, suggesting that maternally inherited *oct4* plays a similarly important role in successful MZT and survival during embryonic development in eel. Transcript levels of *oct4* in later embryonic stages were linked to higher hatch rates in both treatments, which indicates that *oct4* assumes an additional role during later embryonic development and successful hatching.

#### Immune response

During early development, teleost fish rely exclusively on their innate immune system, until their adaptive immune system is sufficiently developed [106]. Maternal transfer of immune-related factors to the eggs has been postulated for several species. While such factors are probably involved in early protection of the embryo, the exact mechanisms are still unknown [28–30]. In sea bream (*Sparus aurata*) [107] and Indian major carp (*Labeo rohita*) [108], maternally transferred *igm* has been associated with higher larval survival. Maternal antibody transfer improved the protection against pathogenic attacks for developing embryos of zebrafish [109]. In European eel, the molecular ontogeny of the immune system has been studied for larval stages from hatch until first-feeding at different temperatures [74], with an array of candidate genes being involved in early immune response. Several of these genes were also investigated in this study, and indicate the maternal transfer of *igm* mRNA in European eel via the ovary to the eggs. Higher mRNA levels in embryos obtained from SPE treated females might indicate a strengthened immune-readiness, due to maternally derived immune factors. Our results showed maternal transfer of *c3* and *il1β* transcripts. While *il1β* exhibited relatively stable abundance throughout embryonic development, *c3* followed a different pattern increasing towards hatch, indicating a potential role for early larval stages.

#### mRNA transcript profiles and MZT

Overall, the mRNA abundance of 20 genes revealed three main patterns of differential expression throughout development. In the first pattern, high abundance in early embryonic development (until 8 hpf) was observed, followed by a drop in mRNA levels after the MZT (between 8 and 24 hpf), likely demonstrating transfer and subsequent clearance of maternal mRNA. This pattern was found for *cldnd*, *foxr1*, *cea*, *ccna1*, *ccnb1*, *ccnb2*, *zar1*, *oct4*, *npm2*, which all are genes involved in early developmental functions, such as cell adhesion, cell division, cell cycle control, oocyte-embryo transition, and MZT activation. This pattern compares to maternal-effect genes known from other teleost species [24–27]. In the second pattern, low mRNA levels were observed during the early embryonic stages (until 8 hpf), with an increase after 8 to 24 hpf, probably demonstrating the activation of zygotic transcription. This was the case for *sox2*, *neurod4*, *neurog1*, *phb2*, and *c3*, primarily genes involved in neurogenesis but also in cell signaling and immune response. This pattern of starting transcription during MZT is common for genes involved in processes such as organogenesis [26]. In the third pattern, changes in transcription throughout development were less prominent including *cdhr2*, *epcam*, *dicer1*, *igm*, *il1β*, genes involved in cell adhesion, microRNA regulation, and immune response. It is notable that a deviating pattern between treatments was observed for *dcbld1* transcripts, which appeared to follow the maternal-effect gene pattern only for embryos obtained from SPE treated females.

Genes whose transcripts followed pattern one included c*ldnd*, a member of the family of claudins, which are known for their importance in generating tight junctions between cells in teleosts [110–112]. C*ldnd* mRNA was found to be highly abundant during early embryonic development in zebrafish [93]. This was also the case in the present study, with highest levels produced by CPE treatment. Ovarian-specific expression of *foxr1* has been shown in zebrafish [113], freshwater medaka (*Oryzias melastigma*) [114], rice field eel (*Monopterus albus*) [115], and European eel [73] possibly indicating an important role during early life history. The vital importance of *foxr1* transcript abundance for embryonic survival around MZT was recently suggested for zebrafish [113], which is consistent with our observed high transcript levels. The family of cyclins is essential for early cell cycle progression in teleosts and highly expressed in early embryonic stages [24,93,116,117]. In rainbow trout (*Oncorhynchus mykiss*), abundance of *ccna1* might be linked to embryonic developmental competence [118]. In this study, mRNA abundance of this gene was not associated with embryonic survival, however gene expression levels at later stages were related to hatch success, which indicates an additional function during later embryonic development in European eel. Lastly, mRNA abundance patterns of *zar1*, first shown to be critical for the oocyte-to-embryo transition in mice [119], also appear to play an important role in early development of rainbow trout [118,120], and Atlantic cod [24].

Within the second pattern of mRNA abundance, *sox2* has been shown to be responsible for the successful activation of the MZT in zebrafish, together with *oct4* and *nanog* [103]. The expression patterns of *sox2* in our study are in accordance with this, indicating a similar function in European eel. For *phb2*, a previous study on European eel found that the abundance of transcripts was higher in a “high hatch group” compared to a “low hatch group” [20]. Our results are similar, providing further support for an important role of this gene during embryonic development in European eel. In contrast, mRNA abundance of this gene in rainbow trout has been negatively correlated with developmental success, indicating species-specific differences [121]. Another important process during embryonic development is neurogenesis, which in teleosts mainly has been studied in zebrafish, where *neurod4* and *neurog1* are key players [33–35]. In our study, their mRNA abundance patterns suggested that they are already important during early development. Interestingly, a negative relationship was found between the mRNA abundance of *neurod4* and *neurog1* during late embryonic development and hatch success, which calls for more detailed research on the function of these two genes during eel embryogenesis.

Representing the third pattern, *dicer1* has been ascribed an essential role in microRNA (miRNA) synthesis during embryonic development. It has mainly been investigated in zebrafish [10,122], where lack of *dicer1* transcripts led to slower growth rates and shorter survival [123], as well as abnormal persistence of maternal mRNA beyond MZT [13,124]. High levels of *dicer1* transcripts in rainbow trout embryos suggest an important role during early development [125]. This is in accordance with our results, which show high levels and importance before MZT with a possible association with the occurrence of cleavage abnormalities.

The association between mRNA levels at 2 hpf of three genes, *zar1*, *epcam*, *dicer1* and cleavage abnormalities as well as between mRNA levels before MZT (8 hpf) of seven genes, *zar1*, *sox2*, *foxr1*, *cldnd*, *phb2*, *neurod4*, *neurog1* and later embryonic survival (48 hpf) indicated maternal mRNA transfer and the importance of all these genes for successful development in European eel embryos. The expression of five genes, *ccna1*, *npm2*, *oct4*, *neurod4*, *neurog1* during later embryonic development (32 hpf) was associated with hatch success and corroborated the importance of their transcription during the transition from maternal mRNA control to zygotic transcription.

## Conclusion

Assisted reproduction protocols developed for anguillid eels typically use repeated administration of pituitary extract for induction of vitellogenesis. Ours is the first study that compared differences in egg quality and embryonic developmental competence between carp and salmon pituitary extracts. In two successive trials, using a constant weekly dose, a higher proportion of female broodstock responded to CPE treatment, however a higher proportion of SPE treated females produced viable offspring. The lower embryonic developmental success of CPE treated females was associated with abnormalities in cell cleavages during early embryogenesis. These findings point to differences in constituents of the carp and salmon pituitaries applied, affecting female response, oocyte development during vitellogenesis, and final maturation. Complementing gene expression analyses showed that differences in embryonic survival were related to differences in mRNA transcript abundance of eight genes involved in cell adhesion, cell division, cell cycle control, MZT activation, and immune regulation. Thus, the differential impact of CPE and SPE appeared to be related to the variability in mRNA abundance in the eggs including maternal transcripts known to be important for healthy embryonic development. Differential expression patterns during embryonic development were observed for 20 genes involved in key mechanisms showing either increasing or decreasing expression profiles around the MZT. The mechanisms regulating the transfer of mRNA to the developing oocytes are still poorly known, however follicular development may be affected by differences in content, composition or affinity of gonadotropins, FSH and LH, as well as other pituitary hormones affecting the deposition of mRNA transcripts into the oocytes. Unravelling the influences of hormonal factors in PEs may prove important to developing novel treatment protocols. Better understanding of the physiology and ontogeny of maternal and embryonic mRNA transcript abundance of different genes during embryogenesis will alleviate early development failure in teleost species in aquaculture.

## References

[1] Teletchea F. Domestication of Marine Fish Species: Update and Perspectives. J Mar Sci Eng. 2015;3: 1227–1243.

[2] Metian M, Troell M, Christensen V, Steenbeek J, Pouil S. Mapping diversity of species in global aquaculture. Rev Aquac. 2019; 1–11.

[3] Kjørsvik E, Mangor-Jensen A, Homefjord I. Egg quality in marine fishes. Adv Mar Biol. 1990;26: 71–113.

[4] Brooks S, Tyler CR, Sumpter JP. Egg quality in fish: what makes a good egg? Rev Fish Biol Fish. 1997;7: 387–416.

[5] Bobe J, Labbé C. Egg and sperm quality in fish. Gen Comp Endocrinol. 2010;165: 535–548. 10.1016/j.ygcen.2009.02.011 19272390

[6] Mylonas CC, Fostier A, Zanuy S. Broodstock management and hormonal manipulations of fish reproduction. Gen Comp Endocrinol. 2010;165: 516–534. 10.1016/j.ygcen.2009.03.007 19318108

[7] Lubzens E, Bobe J, Young G, Sullivan CV. Maternal investment in fish oocytes and eggs: The molecular cargo and its contributions to fertility and early development. Aquaculture. 2017;472: 107–143.

[8] Migaud H, Bell G, Cabrita E, McAndrew B, Davie A, Bobe J, et al Gamete Quality and Broodstock Management in Temperate Fish. In: Conceição LEC, Tandler A, editors. Success factors for fish larval production. Wiley-Blackwell; 2018 pp. 3–39.

[9] Sullivan C V., Chapman RW, Reading BJ, Anderson PE. Transcriptomics of mRNA and egg quality in farmed fish: Some recent developments and future directions. Gen Comp Endocrinol. 2015;221: 23–30. 10.1016/j.ygcen.2015.02.012 25725305

[10] Abrams EW, Mullins MC. Early zebrafish development: It’s in the maternal genes. Curr Opin Genet Dev. 2009;19: 396–403. 10.1016/j.gde.2009.06.002 19608405 PMC2752143

[11] Newport J, Kirschner M. A major developmental transition in early xenopus embryos: I. characterization and timing of cellular changes at the midblastula stage. Cell. 1982;30: 675–686. 10.1016/0092-8674(82)90272-0 6183003

[12] Tadros W, Lipshitz HD. The maternal-to-zygotic transition: a play in two acts. Development. 2009;136: 3033–3042. 10.1242/dev.033183 19700615

[13] Giraldez AJ, Mishima Y, Rihel J, Grocock RJ, Dongen S Van, Inoue K, et al Deadenylation and Clearance of Maternal mRNAs. Science. 2006;312: 75–80. 10.1126/science.1122689 16484454

[14] Lee MT, Bonneau AR, Giraldez AJ. Zygotic Genome Activation During the Maternal-to-Zygotic Transition. Annu Rev Cell Dev Biol. 2014;30: 581–613. 10.1146/annurev-cellbio-100913-013027 25150012 PMC4303375

[15] Giraldez AJ. MicroRNAs, the cell’s Nepenthe: Clearing the past during the maternal-to-zygotic transition and cellular reprogramming. Curr Opin Genet Dev. 2010;20: 369–375. 10.1016/j.gde.2010.04.003 20452200 PMC2908189

[16] Schier AF. The maternal-zygotic transition: Death and birth of RNAs. Science. 2007;316: 406–407. 10.1126/science.1140693 17446392

[17] Stitzel M, Seydoux G. Regulation of the Oocyte-to-Zygote Transition. Science. 2007;316: 407–408. 10.1126/science.1138236 17446393

[18] Aegerter S, Jalabert B, Bobe J. Messenger RNA Stockpile of Cyclin B, Insulin-Like Growth Factor I, Insulin-Like Growth Factor II, Insulin-Like Growth Factor Receptor Ib, and p53 in the Rainbow Trout Oocyte in Relation with Developmental Competence. Mol Reprod Dev. 2004;67: 127–135. 10.1002/mrd.10384 14694427

[19] Lanes CFC, Bizuayehu TT, de Oliveira Fernandes JM, Kiron V, Babiak I. Transcriptome of Atlantic Cod (*Gadus morhua* L.) Early Embryos from Farmed and Wild Broodstocks. Mar Biotechnol. 2013;15: 677–694. 10.1007/s10126-013-9527-y 23887676

[20] Rozenfeld C, Butts IAE, Tomkiewicz J, Zambonino-Infante JL, Mazurais D. Abundance of specific mRNA transcripts impacts hatching success in European eel, *Anguilla anguilla* L. Comp Biochem Physiol -Part A Mol Integr Physiol. 2016;191: 59–65. 10.1016/j.cbpa.2015.09.011 26415730

[21] Škugor A, Krasnov A, Andersen Ø. Genome-wide microarray analysis of Atlantic cod (*Gadus morhua*) oocyte and embryo. BMC Genomics. 2014;15: 594 10.1186/1471-2164-15-594 25023375 PMC4124161

[22] Mommens M, Fernandes JMO, Bizuayehu TT, Bolla SL, Johnston IA, Babiak I. Maternal gene expression in Atlantic halibut (Hippoglossus hippoglossus L.) and its relation to egg quality. BMC Res Notes. 2010;3. 10.1186/1756-0500-3-138 PMC2897799 20497529

[23] Chapman RW, Reading BJ, Sullivan CV. Ovary transcriptome profiling via artificial intelligence reveals a transcriptomic fingerprint predicting egg quality in striped bass, *Morone saxatilis*. PLoS One. 2014;9. 10.1371/journal.pone.0096818 PMC4018430 24820964

[24] Drivenes Ø, Taranger GL, Edvardsen RB. Gene Expression Profiling of Atlantic Cod (*Gadus morhua*) Embryogenesis Using Microarray. Mar Biotechnol. 2012;14: 167–176. 10.1007/s10126-011-9399-y 21833508

[25] Rise ML, Nash GW, Hall JR, Booman M, Hori TS, Trippel EA, et al Variation in embryonic mortality and maternal transcript expression among Atlantic cod (*Gadus morhua*) broodstock: A functional genomics study. Mar Genomics. 2014;18: 3–20. 10.1016/j.margen.2014.05.004 24878168

[26] Mathavan S, Lee SGP, Mak A, Miller LD, Murthy KRK, Govindarajan KR, et al Transcriptome analysis of zebrafish embryogenesis using microarrays. PLoS Genet. 2005;1: 0260–0276. 10.1371/journal.pgen.0010029 PMC1193535 16132083

[27] Sørhus E, Incardona JP, Furmanek T, Jentoft S, Meier S, Edvardsen RB. Developmental transcriptomics in Atlantic haddock: Illuminating pattern formation and organogenesis in non-model vertebrates. Dev Biol. Elsevier; 2016;411: 301–313. 10.1016/j.ydbio.2016.02.012 26875497

[28] Magnadottir B, Lange S, Gudmundsdottir S, Bøgwald J, Dalmo RA. Ontogeny of humoral immune parameters in fish. Fish Shellfish Immunol. 2005;19: 429–439. 10.1016/j.fsi.2005.03.010 15916905

[29] Mulero I, García-Ayala A, Meseguer J, Mulero V. Maternal transfer of immunity and ontogeny of autologous immunocompetence of fish: A minireview. Aquaculture. 2007;268: 244–250.

[30] Zhang S, Wang Z, Wang H. Maternal immunity in fish. Dev Comp Immunol. 2013;39: 72–78. 10.1016/j.dci.2012.02.009 22387589

[31] Swain P, Nayak SK. Role of maternally derived immunity in fish. Fish Shellfish Immunol. 2009;27: 89–99. 10.1016/j.fsi.2009.04.008 19442742

[32] Lo J, Lee S, Xu M, Liu F, Ruan H, Eun A, et al 15,000 Unique Zebrafish EST Clusters and Their Future Use in Microarray for Profiling Gene Expression Patterns During Embryogenesis. Genome Res. 2003;13: 455–466. 10.1101/gr.885403 12618376 PMC430290

[33] Schmidt R, Strähle U, Scholpp S. Neurogenesis in zebrafish—from embryo to adult. Neural Dev. 2013;8: 1–13. 10.1186/1749-8104-8-1 23433260 PMC3598338

[34] Blader P, Plessy C, Straehle U. Multiple regulatory elements with spatially and temporally distinct activities control neurogenin1 expression in primary neurons of the zebrafish embryo. Mech Dev. 2003;120: 211–218. 10.1016/s0925-4773(02)00413-6 12559493

[35] Madelaine R, Garric L, Blader P. Partially redundant proneural function reveals the importance of timing during zebrafish olfactory neurogenesis. Development. 2011;138: 4753–4762. 10.1242/dev.066563 21965609

[36] Nagahama Y, Yamashita M. Regulation of oocyte maturation in fish. Dev Growth Differ. 2008;50: 195–219. 10.1111/j.1440-169X.2008.01019.x 18482399

[37] Vidal B, Pasqualini C, Le Belle N, Claire M, Holland H, Sbaihi M, et al Dopamine Inhibits Luteinizing Hormone Synthesis and Release in the Juvenile European Eel: A Neuroendocrine Lock for the Onset of Puberty. Biol Reprod. 2004;71: 1491–1500. 10.1095/biolreprod.104.030627 15229141

[38] Mylonas C, Zohar Y. Controlling fish reproduction in aquaculture. New Technologies in Aquaculture: Improving Production Efficiency, Quality and Environmental Management. Woodhead Publishing Series in Food Science, Technology and Nutrition; 2009 pp. 109–142.

[39] Butts IAE, Sørensen SR, Politis SN, Tomkiewicz J. First-feeding by European eel larvae: A step towards closing the life cycle in captivity. Aquaculture. 2016;464: 451–458.

[40] Politis SN, Sørensen SR, Mazurais D, Servili A, Zambonino-Infante JL, Miest JJ, et al Molecular ontogeny of first-feeding Euopean eel larvae. Front Physiol. 2018;9: 1–15. 10.3389/fphys.2018.00001 30459634 PMC6232945

[41] Tomkiewicz J, Politis SN, Sørensen SR, Butts IAE, Kottmann JS. European eel–an integrated approach to establish eel hatchery technology in Denmark. In: Don A, Coulson P, editors. Eels Biology, Monitoring, Management, Culture and Exploitation: Proceedings of the First International Eel Science Symposium. 5m Publishing; 2019.

[42] Tanaka H, Kagawa H, Ohta H. Production of leptocephali of Japanese eel (*Anguilla japonica*) in captivity. Aquaculture. 2001;201: 51–60.

[43] Tanaka H, Kagawa H, Ohta H, Unuma T, Nomura K. The first production of glass eel in captivity: Fish reproductive physiology facilitates great progress in aquaculture. Fish Physiol Biochem. 2003;28: 493–497.

[44] Kottmann JS, Tomkiewicz J, Butts IAE, Lund I, Jacobsen C, Støttrup JG, et al Effects of essential fatty acids and feeding regimes on egg and offspring quality of European eel: Comparing reproductive success of farm-raised and wild-caught broodstock. Aquaculture. 2020; Forthcoming.

[45] Rousseau K, Lafont A-G, Maugars G, Jolly C, Sébert M-E, Aroua S, et al Advances in Eel Reproductive Physiology and Endocrinology. In: Trischitta F, Takei Y, Sébert P, editors. Eel Physiology. Boca Raton, Fl. USA: CRS Press; 2009 pp. 1–43.

[46] Bezdenezhnykh VA. Obtaining the larvae of European eel *Anguilla anguilla* L.(Pisces, Anguillidae) under experimental conditions. Dokl Akad Nauk SSSR. 1983 pp. 1264–1266.

[47] Yamamoto J, Yamauchi K. Sexual maturation of Japanese eel and production of eel larvae in the aquarium. Nature. 1974;251: 220–222. 10.1038/251220a0 4417634

[48] Pérez L, Peñaranda DS, Dufour S, Baloche S, Palstra AP, Van Den Thillart GEEJM, et al Influence of temperature regime on endocrine parameters and vitellogenesis during experimental maturation of European eel (*Anguilla anguilla*) females. Gen Comp Endocrinol. 2011;174: 51–59. 10.1016/j.ygcen.2011.08.009 21871894

[49] Palstra AP, Cohen EGH, Niemantsverdriet PRW, Van Ginneken VJT, Van Den Thillart GEEJM. Artificial maturation and reproduction of European silver eel: Development of oocytes during final maturation. Aquaculture. 2005;249: 533–547.

[50] Mordenti O, Di Biase A, Sirri R, Modugno S, Tasselli A. Induction of Sexual Maturation in Wild Female European Eels (*Anguilla anguilla*) in Darkness and Light. Isr J Aquac. 2012;

[51] Di Biase A, Casalini A, Emmanuele P, Mandelli M, Lokman PM, Mordenti O. Controlled reproduction in *Anguilla anguilla* (L.): comparison between spontaneous spawning and stripping-insemination approaches. Aquac Res. 2016;47: 3052–3060.

[52] Ohta H, Kagawa H, Tanaka H, Okuzawa K, Iinuma N, Hirose K. Artificial induction of maturation and fertilization in the Japanese eel, *Anguilla japonica*. Fish Physiol Biochem. 1997;17: 163–169.

[53] Oliveira K, Hable WE. Artificial maturation, fertilization, and early development of the American eel (*Anguilla rostrata*). Can J Zool. 2010;88: 1121–1128.

[54] Lokman PM, Young G. Induced spawning and early ontogeny of New Zealand freshwater eels (*Anguilla dieffenbachii* and *A*. *australis*). New Zeal J Mar Freshw Res. 2000;34: 135–145.

[55] Lokman PM, Wylie MJ, Downes M, Di Biase A, Damsteegt EL. Artificial induction of maturation in female silver eels, *Anguilla australis*: The benefits of androgen pre-treatment. Aquaculture. 2015;437: 111–119.

[56] Satoh H, Yamamori K, Hibiya T. Induced Spawning of the Japanese Eel. Nippon Suisan Gakkaishi. 1992;58: 825–832.

[57] da Silva FG, Støttrup J, Kjørsvik E, Tveiten H, Tomkiewicz J. Interactive effects of dietary composition and hormonal treatment on reproductive development of cultured female European eel, *Anguilla anguilla*. Anim Reprod Sci. 2016;171: 17–26. 10.1016/j.anireprosci.2016.05.007 27264530

[58] Tomkiewicz J (Ed. Reproduction of European Eel in Aquaculture (REEL) Consolidation and new production methods. DTU Aqua Report 249–2012. 2012.

[59] Ohta H, Kagawa H, Tanaka H, Okuzawa K, Hirose K. Changes in fertilization and hatching rates with time after ovulation induced by 17, 20[beta]-dihydroxy-4-pregnen-3-one in the Japanese eel, *Anguilla japonica*. Aquaculture. 1996;139: 291–301.

[60] da Silva FFG, Jacobsen C, Kjørsvik E, G. Støttrup J, Tomkiewicz J. Oocyte and egg quality indicators in European eel: Lipid droplet coalescence and fatty acid composition. Aquaculture. 2018;496: 30–38.

[61] Sørensen SR, Gallego V, Pérez L, Butts IAE, Tomkiewicz J, Asturiano JF. Evaluation of methods to determine sperm density for the European eel, *Anguilla anguilla*. Reprod Domest Anim. 2013;48: 936–944. 10.1111/rda.12189 23772654

[62] Peñaranda DS, Pérez L, Gallego V, Barrera R, Jover M, Asturiano JF. European eel sperm diluent for short-term storage. Reprod Domest Anim. 2010;45: 407–415. 10.1111/j.1439-0531.2008.01206.x 18954399

[63] Butts IAE, Sørensen SR, Politis SN, Pitcher TE, Tomkiewicz J. Standardization of fertilization protocols for the European eel, *Anguilla anguilla*. Aquaculture. 2014;426–427: 9–13.

[64] Sørensen SR, Butts IAE, Munk P, Tomkiewicz J. Effects of salinity and sea salt type on egg activation, fertilization, buoyancy and early embryology of European eel, *Anguilla anguilla*. Zygote. 2016;24: 121–138. 10.1017/S0967199414000811 25707438

[65] Politis SN, Mazurais D, Servili A, Zambonino-Infante J-L, Miest JJ, Sørensen SR, et al Temperature effects on gene expression and morphological development of European eel, *Anguilla anguilla* larvae. PLoS One. 2017;12: e0182726 10.1371/journal.pone.0182726 28806748 PMC5555698

[66] Henkel CV, Burgerhout E, de Wijze DL, Dirks RP, Minegishi Y, Jansen HJ, et al Primitive duplicate hox clusters in the european eel’s genome. PLoS One. 2012;7. 10.1371/journal.pone.0032231 PMC3286462 22384188

[67] Stanke M, Diekhans M, Baertsch R, Haussler D. Using native and syntenically mapped cDNA alignments to improve de novo gene finding. Bioinformatics. 2008;24: 637–644. 10.1093/bioinformatics/btn013 18218656

[68] Götz S, García-Gómez JM, Terol J, Williams TD, Nagaraj SH, Nueda MJ, et al High-throughput functional annotation and data mining with the Blast2GO suite. Nucleic Acids Res. 2008;36: 3420–3435. 10.1093/nar/gkn176 18445632 PMC2425479

[69] Miest JJ, Arndt C, Adamek M, Steinhagen D, Reusch TBH. Dietary β-glucan (MacroGard®) enhances survival of first feeding turbot (*Scophthalmus maximus*) larvae by altering immunity, metabolism and microbiota. Fish Shellfish Immunol. Elsevier Ltd; 2016;48: 94–104. 10.1016/j.fsi.2015.11.013 26564474

[70] Hellemans J, Mortier G, De Paepe A, Speleman F, Vandesompele J. qBase relative quantification framework and software for management and automated analysis of real-time quantitative PCR data. Genome Biol. 2008;8: R19. 10.1186/gb-2007-8-2-r19 PMC1852402 17291332

[71] Livak KJ, Schmittgen TD. Analysis of relative gene expression data using real-time quantitative PCR and the 2 ^-ΔΔCT^ method. Methods. 2001;25: 402–408. 10.1006/meth.2001.1262 11846609

[72] Politis SN, Sørensen SR, Mazurais D, Servili A, Zambonino-Infante JL, Miest JJ, et al Molecular ontogeny of first-feeding european eel larvae. Front Physiol. 2018;9: 1–15. 10.3389/fphys.2018.00001 30459634 PMC6232945

[73] Geffroy B, Guilbaud F, Amilhat E, Beaulaton L, Vignon M, Huchet E, et al Sexually dimorphic gene expressions in eels: Useful markers for early sex assessment in a conservation context. Sci Rep. Nature Publishing Group; 2016;6: 1–11. 10.1038/s41598-016-0001-8 27658729 PMC5034313

[74] Miest JJ, Politis SN, Adamek M, Tomkiewicz J, Butts IAE. Molecular ontogeny of larval immunity in European eel at increasing temperatures. Fish Shellfish Immunol. 2019;87: 105–119. 10.1016/j.fsi.2018.12.048 30590168

[75] Littell R, Milliken G, Stroup W, Wolfinger R. SAS system for mixed models. Cary, North Carolina: SAS Institute Incorporated; 1996.

[76] Yossa R, Verdegem M. Misuse of multiple comparison tests and underuse of contrast procedures in aquaculture publications. Aquaculture. 2015;437: 344–350.

[77] Zhou Y, Niu ÆY, Tao ÆM, Deng ÆX. Molecular cloning, characterization and expression of FSH and LH beta subunits from grass carp (*Ctenopharyngodon idella*). Fish Physiol Biochem. 2010;36: 213–221. 10.1007/s10695-008-9223-4 18777101

[78] Kobayashi T, Pakarinen P, Torgersen J, Huhtaniemi I, Andersen Ø. The gonadotropin receptors FSH-R and LH-R of Atlantic halibut (*Hippoglossus hippoglossus*)—2. Differential follicle expression and asynchronous oogenesis. Gen Comp Endocrinol. 2008;156: 595–602. 10.1016/j.ygcen.2008.02.010 18377904

[79] Ramadhani AW, Samik A, Madyawati SP. Vitellogenesis of Giant Gourami (*Osphronemus goramy*) Examined by the Measurement of Estradiol- 17β, Vitellogenin Concentration and the Size of Ovary. Aquac Stud. 2018;18: 113–125.

[80] Vazirzadeh A, Mojazi Amiri B, Fostier A. Ovarian development and related changes in steroid hormones in female wild common carp (*Cyprinus carpio carpio*), from the South-eastern Caspian Sea. J Anim Physiol Anim Nutr (Berl). 2014;98: 1060–1067. 24621281 10.1111/jpn.12171

[81] Palstra AP, Jéhannet P, Swinkels W, Heinsbroek LTN, Lokman PM, Vesala S, et al First Observation of a Spontaneously Matured Female European Eel (*Anguilla anguilla*). Sci Rep. 2020;10: 1–6. 10.1038/s41598-019-56847-4 32047193 PMC7012921

[82] Suetake H, Okubo K, Sato N, Yoshiura Y, Suzuki Y, Aida K. Differential expression of two gonadotropin (GTH) β subunit genes during ovarian maturation induced by repeated injection of salmon GTH in the Japanese eel *Anguilla japonica*. Fish Sci. 2002;68: 290–298.

[83] Yaron Z, Gur G, Melamed P, Rosenfeld H, Elizur A, Levavi-Sivan B. Regulation of Fish Gonadotropins. Int Rev Cytol. 2003;226: 131–185. 10.1016/s0074-7696(05)25004-0 12696592

[84] Schmitz M, Aroua S, Vidal B, Le Belle N, Elie P, Dufour S. Differential regulation of luteinizing hormone and follicle-stimulating hormone expression during ovarian development and under sexual steroid feedback in the European eel. Neuroendocrinology. 2005;81: 107–119. 10.1159/000086404 15961957

[85] Avery TS, Killen SS, Hollinger TR. The relationship of embryonic development, mortality, hatching success, and larval quality to normal or abnormal early embryonic cleavage in Atlantic cod, *Gadus morhua*. Aquaculture. 2009;289: 265–273.

[86] Avery TS, Brown JA. Investigating the relationship among abnormal patterns of cell cleavage, egg mortality and early larval condition in *Limanda ferruginea*. J Fish Biol. 2005;67: 890–896.

[87] Kjørsvik E, Hoehne-Reitan K, Reitan KI. Egg and larval quality criteria as predictive measures for juvenile production in turbot (*Scophthalmus maximus* L.). Aquaculture. 2003;227: 9–20.

[88] Schmoker AM, Ebert AM, Ballif BA. The DCBLD receptor family: Emerging signaling roles in development, homeostasis and disease. Biochem J. 2019;476: 931–950. 10.1042/BCJ20190022 30902898

[89] Slanchev K, Carney TJ, Stemmler MP, Koschorz B, Amsterdam A, Schwarz H, et al The epithelial cell adhesion molecule EpCAM is required for epithelial morphogenesis and integrity during zebrafish epiboly and skin development. PLoS Genet. 2009;5. 10.1371/journal.pgen.1000563 PMC2700972 19609345

[90] Trzpis M. EpCAM in morphogenesis. Front Biosci. 2008;13: 5050–5055. 10.2741/3063 18508569

[91] Trzpis M, McLaughlin PMJ, De Leij LMFH, Harmsen MC. Epithelial cell adhesion molecule: More than a carcinoma marker and adhesion molecule. Am J Pathol. American Society for Investigative Pathology; 2007;171: 386–395. 10.2353/ajpath.2007.070152 17600130 PMC1934518

[92] Lanes CFC, Bizuayehu TT, de Oliveira Fernandes JM, Kiron V, Babiak I. Transcriptome of Atlantic Cod (*Gadus morhua L*.) Early Embryos from Farmed and Wild Broodstocks. Mar Biotechnol. 2013;15: 677–694. 10.1007/s10126-013-9527-y 23887676

[93] Vesterlund L, Hong J, Unneberg P, Hovatta O, Kere J. The zebrafish transcriptome during early development. Bmc Dev Biol. 2011;11: 30 10.1186/1471-213X-11-30 21609443 PMC3118190

[94] Villablanca EJ, Renucci A, Sapède D, Lec V, Soubiran F, Sandoval PC, et al Control of cell migration in the zebrafish lateral line: Implication of the gene “tumour-associated calcium signal transducer,” tacstd. Dev Dyn. 2006;235: 1578–1588. 10.1002/dvdy.20743 16552761

[95] Froschauer A, Khatun MM, Sprott D, Franz A, Rieger C, Pfennig F, et al oct4-EGFP reporter gene expression marks the stem cells in embryonic development and in adult gonads of transgenic medaka. Mol Reprod Dev. 2013;80: 48–58. 10.1002/mrd.22135 23139203

[96] Wang D, Manali D, Wang T, Bhat N, Hong N, Li Z, et al Identification of pluripotency genes in the fish medaka. Int J Biol Sci. 2011;7: 440–451. 10.7150/ijbs.7.440 21547061 PMC3088286

[97] Sánchez-Sánchez A V., Camp E, García-España A, Leal-Tassias A, Mullor JL. Medaka Oct4 is expressed during early embryo development, and in primordial germ cells and adult gonads. Dev Dyn. 2010;239: 672–679. 10.1002/dvdy.22198 20034054

[98] Liu R, Li M, Li Z, Hong N, Xu H, Hong Y. Medaka Oct4 is Essential for Pluripotency in Blastula Formation and ES Cell Derivation. Stem Cell Rev Reports. 2015;11: 11–23. 10.1007/s12015-014-9523-2 25142379

[99] Jing W, Xiaohuan H, Zhenhua F, Zhuo Y, Fan D, Wenjing T, et al Promoter activity and regulation of the Pou5f1 homolog from a teleost, Nile tilapia. Gene. 2018;642: 277–283. 10.1016/j.gene.2017.11.036 29155325

[100] Xiaohuan H, Yang Z, Linyan L, Zhenhua F, Linyan Z, Zhijian W, et al Characterization of the POU5F1 Homologue in Nile Tilapia: From Expression Pattern to Biological Activity. Stem Cells Dev. 2016;25: 1386–1395. 10.1089/scd.2016.0143 27473876

[101] Lachnit M, Kur E, Driever W. Alterations of the cytoskeleton in all three embryonic lineages contribute to the epiboly defect of Pou5f1/Oct4 deficient MZspg zebrafish embryos. Dev Biol. 2008;315: 1–17. 10.1016/j.ydbio.2007.10.008 18215655

[102] Burgess S, Reim G, Chen W, Hopkins N, Brand M. The zebrafish spiel-ohne-grenzen (spg) gene encodes the POU domain protein Pou2 related to mammalian Oct4 and is essential for formation of the midbrain and hindbrain, and for pre-gastrula morphogenesis. Development. 2002;129: 905–916. 11861474 10.1242/dev.129.4.905

[103] Lee MT, Bonneau AR, Takacs CM, Bazzini AA, DiVito KR, Fleming ES, et al Nanog, Pou5f1 and SoxB1 activate zygotic gene expression during the maternal-to-zygotic transition. Nat Rev Drug Discov. 2013;503: 360–364. 10.1038/nature12632 PMC3925760 24056933

[104] Onichtchouk D, Geier F, Polok B, Messerschmidt DM, Mössner R, Wendik B, et al Zebrafish Pou5f1-dependent transcriptional networks in temporal control of early development. Mol Syst Biol. 2010;6. 10.1038/msb.2010.9 PMC2858445 20212526

[105] Reim G, Brand M. Maternal control of vertebrate dorsoventral axis formation and epiboly by the POU domain protein Spg/Pou2/Oct4. Development. 2006;133: 2757–2770. 10.1242/dev.02391 16775002

[106] Uribe C, Folch H, Enriquez R, Moran G. Innate and adaptive immunity in teleost fish: A review. Vet Med (Praha). 2011;56: 486–503.

[107] Hanif A, Bakopoulos V, Leonardos I, Dimitriadis GJ. The effect of sea bream (*Sparus aurata*) broodstock and larval vaccination on the susceptibility by Photobacterium damsela subsp. piscicida and on the humoral immune parameters. Fish Shellfish Immunol. 2005;19: 345–361. 10.1016/j.fsi.2004.12.009 15863015

[108] Swain P, Dash S, Bal J, Routray P, Sahoo PK, Sahoo SK, et al Passive transfer of maternal antibodies and their existence in eggs, larvae and fry of Indian major carp, *Labeo rohita* (Ham.). Fish Shellfish Immunol. 2006;20: 519–527. 10.1016/j.fsi.2005.06.011 16157486

[109] Wang H, Ji D, Shao J, Zhang S. Maternal transfer and protective role of antibodies in zebrafish *Danio rerio*. Mol Immunol. 2012;51: 332–336. 10.1016/j.molimm.2012.04.003 22551698

[110] Gupta IR, Ryan AK. Claudins: Unlocking the code to tight junction function during embryogenesis and in disease. Clin Genet. 2010;77: 314–325. 10.1111/j.1399-0004.2010.01397.x 20447145

[111] Kolosov D, Bui P, Chasiotis H, Kelly SP. Claudins in teleost fishes. Tissue Barriers. 2013;1: e25391 10.4161/tisb.25391 24665402 PMC3875606

[112] Günzel D. Claudins: vital partners in transcellular and paracellular transport coupling. Pflugers Arch—Eur J Physiol. 2017;469: 35–44. 27888337 10.1007/s00424-016-1909-3

[113] Cheung CT, Patinote A, Guiguen Y, Bobe J. Foxr1 Is a Novel Maternal-Effect Gene in Fish That Is Required for Early Embryonic Success. PeerJ. 2018;6: e5534 10.7717/peerj.5534 30155373 PMC6109588

[114] Lai KP, Li JW, Wang SY, Chiu JMY, Tse A, Lau K, et al Tissue-specific transcriptome assemblies of the marine medaka Oryzias melastigma and comparative analysis with the freshwater medaka *Oryzias latipes*. BMC Genomics. 2015;16: 1–14. 10.1186/1471-2164-16-1 25765076 PMC4352242

[115] Chi W, Gao Y, Hu Q, Guo W, Li D. Genome-wide analysis of brain and gonad transcripts reveals changes of key sex reversal-related genes expression and signaling pathways in three stages of *Monopterus albus*. PLoS One. 2017;12: e0173974 10.1371/journal.pone.0173974 28319194 PMC5358790

[116] Kreutzer MA, Richards JP, De Silva-Udawatta MN, Temenak JJ, Knoblich JA, Lehner CF, et al Caenorhabditis elegans cyclin A- and B-type genes: A cyclin A multigene family, an ancestral cyclin B3 and differential germline expression. J Cell Sci. 1995;108: 2415–2424. 7545687 10.1242/jcs.108.6.2415

[117] Qiu GF, Ramachandra RK, Rexroad CE, Yao J. Molecular characterization and expression profiles of cyclin B1, B2 and Cdc2 kinase during oogenesis and spermatogenesis in rainbow trout (*Oncorhynchus mykiss*). Anim Reprod Sci. 2008;105: 209–225. 10.1016/j.anireprosci.2007.03.005 17399922

[118] Aegerter S, Jalabert B, Bobe J. Large scale real-time PCR analysis of mRNA abundance in rainbow trout eggs in relationship with egg quality and post-ovulatory ageing. Mol Reprod Dev. 2005;72: 377–385. 10.1002/mrd.20361 16075464

[119] Wu X, Viveiros MM, Eppig JJ, Bai Y, Fitzpatrick SL, Matzuk MM. Zygote arrest 1 (Zar1) is a novel maternal-effect gene critical for the oocyte-to-embryo transition. Nat Genet. 2003;33: 187–191. 10.1038/ng1079 12539046

[120] Bobe J, Nguyen T, Mahé S, Monget P. In silico identification and molecular characterization of genes predominantly expressed in the fish oocyte. BMC Genomics. 2008;9: 1–16. 10.1186/1471-2164-9-1 18947432 PMC2584112

[121] Bonnet E, Fostier A, Bobe J. Microarray-based analysis of fish egg quality after natural or controlled ovulation. BMC Genomics. 2007;8: 55 10.1186/1471-2164-8-55 17313677 PMC1808064

[122] Bizuayehu TT, Babiak I. MicroRNA in teleost fish. Genome Biol Evol. 2014;6: 1911–1937. 10.1093/gbe/evu151 25053657 PMC4159003

[123] Wienholds E, Koudijs MJ, Van Eeden FJM, Cuppen E, Plasterk RHA. The microRNA-producing enzyme Dicer1 is essential for zebrafish development. Nat Genet. 2003;35: 217–218. 10.1038/ng1251 14528306

[124] Giraldez AJ. MicroRNAs regulate brain morphogenesis in zebrafish. Science. 2005;2196. 10.1126/science.1109020 15774722

[125] Ramachandra RK, Salem M, Gahr S, Rexroad CE, Yao J. Cloning and characterization of microRNAs from rainbow trout (*Oncorhynchus mykiss*): Their expression during early embryonic development. BMC Dev Biol. 2008;8: 1–11. 10.1186/1471-213X-8-1 18412968 PMC2374770

